# 978. Association of oral anticoagulation therapy with dabigatran and the incidence rate of Staphylococcus aureus bacteremia among patients with atrial fibrillation

**DOI:** 10.1093/ofid/ofad500.033

**Published:** 2023-11-27

**Authors:** Shinya Hasegawa, Michael Ohl, Mary Vaughan-Sarrazin, Eli N Perencevich, Michihiko Goto

**Affiliations:** University of Iowa Carver College of Medicine, Iowa City, Iowa; University of Iowa Carver College of Medicine, Iowa City, Iowa; University of Iowa College of Medicine, Iowa City, Iowa; University of Iowa/Iowa City VAMC, Iowa City, Iowa; University of Iowa/Iowa City VAMC, Iowa City, Iowa

## Abstract

**Background:**

Dabigatran, a univalent direct thrombin inhibitor, is used as a direct oral anticoagulant (DOAC) for patients with high-risk conditions for thrombosis, such as atrial fibrillation (AF). In-vitro and animal model studies suggest dabigatran (but not other DOACs) might also reduce the virulence of Staphylococcus aureus by inhibiting staphylothrombin, and a recent study from Denmark reported lower incidence of S aureus bacteremia (SAB) among people on dabigatran compared to other DOACs (Butt et al. CID 2021). However, clinical data for this phenomenon in other populations are still sparse. We aimed to evaluate the incidence rate of SAB among patients in the United States Veterans Health Administration (VHA) treated with dabigatran compared with those treated with other DOACs or warfarin.

**Methods:**

This retrospective cohort study examined medication and microbiology data for all patients with AF who started new oral anticoagulation therapies in VHA in 2011-18 and followed for the duration of the first anticoagulation therapy. We calculated crude incidence rates of SAB as the number of events per 10,000 person-years, stratified by the type of oral anticoagulation therapy.

**Results:**

Among 45,023 patients starting anticoagulation for AF during the study period, the total person-years of exposure were 5,384 for dabigatran (5,052 unique patients), 12,613 for non-dabigatran DOACs (15,449 unique patients), and 38,964 for warfarin (31,415 unique patients). There were 11 SAB events among patients on dabigatran, 52 among patients on non-dabigatran DOACs, and 194 among patients on warfarin. The crude incidence rates of SAB in each group were 20.43 (95% confidence interval [CI], 8.37-32.49), 41.23 (95%CI, 30.04-52.41), and 49.79 (95%CI, 42.80-56.78) events per 10,000 person-years, respectively (dabigatran vs non-dabigatran DOACs: p=0.043, dabigatran vs warfarin: p=0.004).

**Conclusion:**

The crude incidence rate of SAB among Veterans with AF who were on dabigatran was lower than those on other DOACs or warfarin. This supports the prior Danish study reporting lower SAB incidence among patients on dabigatran. Further studies are needed to assess this potential association while accounting for other patient-level factors and longer follow-up durations.

**Disclosures:**

**Michihiko Goto, MD MSCI**, Merck & Co.: Grant/Research Support

